# The Roles of Carcinoembryonic Antigen in Liver Metastasis and Therapeutic Approaches

**DOI:** 10.1155/2017/7521987

**Published:** 2017-05-10

**Authors:** Joo Han Lee, Seong-Wook Lee

**Affiliations:** ^1^Department of Molecular Biology, Dankook University, Yongin 16890, Republic of Korea; ^2^Department of Integrated Life Sciences, Research Institute of Advanced Omics, Dankook University, Yongin 16890, Republic of Korea

## Abstract

Metastasis is a highly complicated and sequential process in which primary cancer spreads to secondary organic sites. Liver is a well-known metastatic organ from colorectal cancer. Carcinoembryonic antigen (CEA) is expressed in most gastrointestinal, breast, and lung cancer cells. Overexpression of CEA is closely associated with liver metastasis, which is the main cause of death from colorectal cancer. CEA is widely used as a diagnostic and prognostic tumor marker in cancer patients. It affects many steps of liver metastasis from colorectal cancer cells. CEA inhibits circulating cancer cell death. CEA also binds to heterogeneous nuclear RNA binding protein M4 (hnRNP M4), a Kupffer cell receptor protein, and activates Kupffer cells to secrete various cytokines that change the microenvironments for the survival of colorectal cancer cells in the liver. CEA also activates cell adhesion-related molecules. The close correlation between CEA and cancer has spurred the exploration of many CEA-targeted approaches as anticancer therapeutics. Understanding the detailed functions and mechanisms of CEA in liver metastasis will provide great opportunities for the improvement of anticancer approaches against colorectal cancers. In this report, the roles of CEA in liver metastasis and CEA-targeting anticancer modalities are reviewed.

## 1. Introduction

Colorectal cancer (CRC) is a health problem in most industrialized countries worldwide. Globally, it is the third most common cause of cancer-related deaths [1, 2]. According to the World Cancer Research Fund International (http://www.wcrf.org), approximately 1.4 million new cases of CRC were diagnosed in 2012. CRC is diagnosed in nearly 10% of all cancers following lung cancer (13%) and breast cancer (12%) and is the third common cancer in men and the second common cancer in women. South Korea has the highest diagnosed rate of CRC (45 persons per million), followed by Slovakia (42.7 persons per million) and Hungary (42.3 persons per million). About 54% of cases occur in more developed countries. The highest incidence of CRC is reported in Oceania and Europe, while the lowest incidence is in Africa and Asia. The rates of incidence and diagnosis of CRC have gradually increased because of a change in the dietary habits and the increasing prevalence of obesity and smoking [3–5]. The main cause of CRC-related death is liver metastasis, which occurs in 20 to 70% of patients depending on cancer progression [6]. Only a small portion of liver metastases are manageable with current therapeutic treatments.

Carcinoembryonic antigen (CEA, also known as CEACAM5 or CD66e) was discovered in malignant tumors of endodermally derived epithelium of the gastrointestinal tract and pancreas [7]. Since the discovery of CEA nearly five decades ago, it has been revealed to be overexpressed in the majority of human carcinomas [7, 8]. CEA has immunoglobulin-like structural characteristics and many glycosylation modification sites [9]. The close relationship between CRC and CEA expression has prompted the use of CEA as a tumor marker [10, 11]. Measurement of CEA level in serum is clinically useful and reliable for the CRC diagnosis. Elevation in the level of CEA is a prognostic indicator for the state of CRC patients [12, 13]. In CRC, the principle site of metastasis is the liver [14]. CEA overexpression is associated with liver metastasis [15, 16]. CEA also aids multiple steps of CRC-related liver metastasis [15, 16]. Particularly, five amino acids (Pro-Glu-Leu-Pro-Lys, PELPK) existing between the N and A1 domain of CEA are critical in liver metastasis [17, 18].

CEA affects liver metastasis mainly by three steps. In the first step, CEA protects circulating colon cancer cells from death in blood [19–21]. When cells are detached from tissues, anoikis-mediated cell death is induced. However, CEA can prevent circulating cell death through inhibiting anoikis. In the second step, CEA binds to heterogeneous nuclear RNA binding protein M4 (hnRNP M4), a Kupffer cell receptor protein [17, 22–23]. Kupffer cells are macrophages that protect the liver. Following the CEA binding to hnRNP M4, Kupffer cells change the liver microenvironments to favor CRC cells, which increases the likelihood of metastasis [24–27]. In the third step, CEA upregulates cell adhesion molecules for metastasis [9].

Although a plethora of experimental and clinical data have documented the important roles of CEA in liver metastasis from CRC cells, the detailed mechanisms of CEA-mediated liver metastasis remain to be elucidated. Owing to the close relationship between CEA and liver metastasis, various therapeutic approaches that can block the function of CEA have been attempted. This review will focus on the current knowledge of the CEA-mediated regulation of liver metastatic steps and CEA-targeted approaches for cancer therapy.

## 2. CEA

CEA is a member of the immunoglobulin (Ig) superfamily of proteins. The human CEA gene family contains 29 genes/pseudogenes, of which 18 are expressed [28–30]. Several genes in the CEA gene family are also expressed in other mammals including mice, rats, and dogs. The CEA gene family can be divided into three groups based on the sequence similarities and functions: the CEA-related cell adhesion molecule (CEACAM) group, pregnancy-specific glycoprotein (PSG) group, and pseudogene group [31].

**Figure 1:**
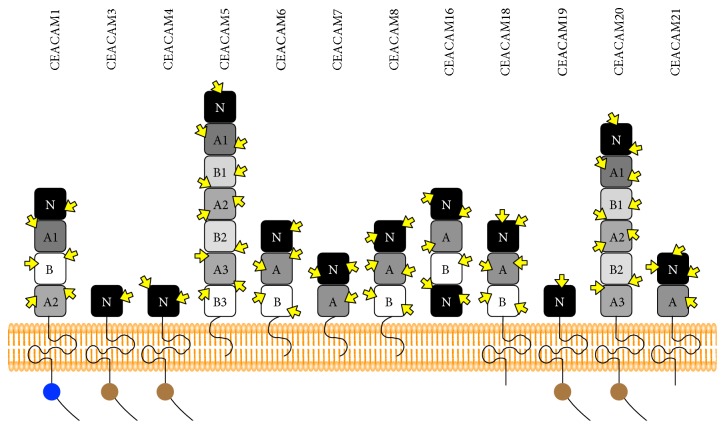
Schematic representation of CEACAM group members. CEACAM1, CEACAM3, CEACAM4, CEACAM19, CEACAM20, and CEACAM21 have transmembrane domains while CEACAM5 (CEA), CEACAM6, CEACAM7, and CEACAM8 have GPI-linked membrane-anchoring characteristics. CEACAM3, CEACAM4, CEACAM19, and CEACAM20 have immunoreceptor tyrosine-based activation motif (ITAM). However, only CEACAM1 has immunoreceptor tyrosine-based inhibition motif (ITIM). Brown circles represent ITAM. Blue circle shows ITIM. CEACAM group members have many glycosylation sites, which are indicated by the yellow arrows.

The CEACAM group consists of a single N-terminal domain and a maximum of six disulfide-linked internal domains. The group contains 12 proteins (CEACAM1, 3–8, 16, 18–21) (Figure 1()). Their N-terminal domain is similar to the antigen recognition domain of Ig. Other domains of the CEACAM group are similar to C2-type Ig domains [31]. The extracellular domains of the CEACAM group function as homophilic and heterophilic cellular adhesion molecules or receptors [32–34]. Members of the CEACAM group might act as dimers or oligomers with other membrane molecules that have diverse functions [9, 35, 36]. CEACAM1, CEA (CEACAM5), and CEACAM6 have been studied concerning cancer progression [32, 37, 38]. Unlike CEA and CEACAM6, CEACAM1 harbors a transmembrane domain and has alternative splicing variants. The expression ratio of the CEACAM1 long (CEACAM1-L) and short (CEACAM1-S) isoform is related with tumorigenesis [39–42].

Liver metastasis has been most closely related to CEA. The molecular weight of CEA protein in normal cells is 72 kDa. However, CEA with a molecular weight of about 180 to 200 kDa has been detected in cancer cell lines and patients, reflecting the numerous glycosylation modification sites and the differing glycosylation patters in cancer cells [8, 43]. Other modification sites besides glycosylation have not been reported. CEA is a glycophosphatidylinositol- (GPI-) linked membrane-anchoring protein that is exposed to the cell surface that faces the extracellular matrix. The membrane-anchoring region of CEA can be cleaved by phospholipase C and phospholipase D. The cleaved products are soluble and circulating through blood vessels [44]. Thus, CEA can be present as secreted and cell surface-anchored forms.

CEA is functionally associated with cellular interaction, cell adhesion, immune response, anoikis resistance, and promotion of liver metastasis [9, 19, 45–47]. CEA overexpression is associated with many types of cancers including gastrointestinal, respiratory, and genitourinary system and breast cancers [8]. CEA is present in the apical membrane of normal tissue but is overexpressed in CRC and occupies the entire surface of cell membranes in colorectal cancer patients [8, 48].

CEA is one of the oldest and most widely used tumor markers for monitoring tumor recurrence after surgical resection and prognosis. A small rise in the level of CEA can be predictive of recurrence following curative surgery for CRC up to a year before the onset of clinical symptoms [49]. Advances in novel imaging and targeting techniques have revealed other tumor markers [10]. However, CEA remains the most reliable and sensitive biomarker for CRC. The expression level of CEA in serum is an important factor for staging colon cancers and in decision-making regarding future therapeutic strategies [10, 11]. CEA protein and mRNA expression levels in serum are useful early markers for recurrence in pancreatic cancer and CRC patients. Elevations of serum CEA of 50–60% can occur [50, 51]. CEACAM1, CEACAM6, and NCA-90 are also used prognostically to predict tumor recurrence of breast, lung, and colorectal cancers [42, 52–55].

CRC-related and overexpressed CEA circulates through the blood vessels and enters the liver. There, it likely affects multiple steps of liver metastasis. CEA protects from anoikis-mediated cell death from circulating CRC cells. CEA can also affect lung metastasis. This review will focus on the roles of CEA in the survival of CRC cells in liver tissue and in liver metastasis from colorectal cancer.

## 3. CEA and Liver Metastasis

Metastasis is a multistep process in which malignant cells spread from the original tumor organ site to colonize distant organ sites [56, 57]. The metastatic cascade involves very complicated cell-biological events. To metastasize, cancer cells have to endure stringent stimuli from neighboring environments and pass through a sequence of steps. These steps include local invasion of cancer cells into surrounding extracellular matrix (ECM) and stromal cell layers, intravasation into blood vessels, survival and circulation in blood vessels, arrest at distant organ sites, extravasation into the parenchyma of distant tissues, initial survival within foreign microenvironments, and reinitiation of proliferation at metastatic sites to generate macroscopic and clinically detectable neoplastic growths [58].

Theoretically, circulating cancer cells that originate from the primary cancer can disseminate and survive in a wide variety of secondary tissues and organs. However, metastasis has been reported in only a limited subset of target organs [59]. The host microenvironments might be one of the important factors and major determinants for the survival of cancer cells in foreign tissues. Cancer cells that originate from a specific organ might have preferential targets of metastasis. Major organs for CRC metastasis are the liver and lung, while major organs for breast cancer-mediated metastasis are the bone, lung, liver, and brain [14, 59–61]. Nearly 80% of metastasis that occurs in CRC is directed at the liver. CRC rarely spreads to the bone [61]. During metastasis, cancer cells acquire the ability to change the microenvironment to favor their survival in secondary organs. Inflammatory responses produced by adjacent stromal cells or macrophages recruited by cancer cells are the most important factors for the survival of the cells in foreign tissues and subsequent metastasis [62, 63]. Many immune-related gene expression levels are affected by metastasis.

A direct relationship between CEA production and liver metastatic potential has been documented for human colon cancer cells [14–16]. Injection of CEA into mice prior to injection of weakly metastatic cancer cells can increase liver metastasis of the injected cells [64]. Poorly metastatic colon cancer cell lines can become highly metastatic following transfection with CEA cDNA [65]. Conversely, inhibition of CEA expression can reduce the liver metastatic potentials of CRC cells [20]. CEA has an amino acid block of Pro-Glu-Leu-Pro-Lys (PELPK) located at position 108–112 between the N and A1 domain hinge region of CEA. The PELPK penta-peptide amino acid sequence is the binding motif of CEA for Kupffer cells, which is associated with the initiation of metastasis and the mesenchymal-epithelial transition (MET) of hepatic metastasis from circulating CRC cells [17, 18].

The influences of CEA on liver metastasis include survival of circulating tumor cells in blood vessels, activation of Kupffer cells by binding to hnRNP M4, Kupffer cell membrane protein, altered liver microenvironments, and adhesion and survival of circulating CRC cells in the liver.

### 3.1. Survival of Circulating Tumor Cells by CEA

Most cells except for circulating blood-related cells stay close to tissues. This allows efficient communication between adjacent cells and ECM to provide essential signals for growth and survival. When cells detach from the ECM, they lose the normal cell–matrix interactions and cell polarity. They can undergo anoikis, a process of apoptosis that is induced by detachment of anchorage-dependent cells from the surrounding environments or ECM [66]. Metastasis in distant secondary organs requires tumor cells to overcome anoikis-mediated cell death and survive in blood vessels.

Anoikis-mediated cell death is associated with loss of integrin-mediated cell adhesion signaling [67, 68]. Detached cells can produce tumor necrosis factor- (TNF-) related apoptosis-inducing ligand (TRAIL), the TRAIL-R2 ligand, and death receptor 5 (DR5), a key protein for anoikis in colon cancer cell lines [69]. Cell surface CEA can protect cancer cells from anoikis in CRC patients by directly binding to DR5, thus blocking cell death signals in circulating tumor cells [20]. The PELPK penta-peptide of CEA is also critical in the binding of CEA to DR5; the binding inhibits DR5-mediated downstream cell death signal transductions [70].

Cell surface CEA can also directly interact with transforming growth factor-*β* (TGF-*β*) type I receptor (TBRI). The interaction alters the downstream TGF-*β* signal pathway and increases tumor cell proliferation [71]. Unlike the interaction between CEA and DR5, it is unclear whether the PELPK sequence is involved in the interaction between CEA and TBRI.

CEACAM6 also protects many types of cell lines from apoptosis and anoikis [19, 21, 48, 72]. CEACAM1 is associated with apoptosis in breast and colon cancer cell lines [73–75]. CEA and CEACAM6 are antiapoptotic functional proteins, whereas CEACAM1 participates in apoptosis. The molecular nature of these inverse functions is unclear.

### 3.2. Arrest of Circulating Tumor Cells in the Liver by Binding of CEA to hnRNP M4 in Kupffer Cells and Activation of Kupffer Cells by CEA for Liver Metastasis

In the metastatic cascade, circulating tumor cells are arrested at distant organs. First, they will encounter macrophages produced by differentiation of monocytes in tissues. Both monocytes and macrophages have phagocytic characteristics. The main role of macrophages is phagocytosis, a process of engulfing and digesting cellular debris or pathogens. It eventually protects parenchymal tissues from stimuli and damages. Macrophages also stimulate lymphocytes and other immune cells to respond to pathogens [76]. Kupffer cells are hepatic macrophages located in the hepatic sinusoids through portal circulation. Kupffer cells face the sinusoidal lumen and directly contact the portal circulation. The cells remove chemical compounds and dead or damaged cells, eliminate bacteria, and protect the liver against tumor cells invasion [77]. Elevated levels of circulating CEA secreted from CRC cells can activate Kupffer cell functions, which is a critical step in liver metastasis of CRC cells.

Kupffer cells express hnRNP M4 protein. The protein is a CEA receptor [22] and is ubiquitously expressed. It normally localizes in the nucleus. Kupffer cells, other terminally differentiated macrophages like lung alveolar macrophages, and some cancer cells including human CRC cell line HT29 express hnRNP M4 on the cell surface [23]. What orients hnRNP M4 to the cell surface is unknown. Kupffer cells express two alternative splicing variant forms of hnRNP M4. Both bind to CEA [22]. The major roles of hnRNP are regulating mRNA processing, alternative splicing, microRNA biosynthesis, and mRNA transport to the cytoplasm from the nucleus [78]. In contrast, hnRNP M4 has a unique function in Kupffer cells and lung alveolar macrophages as a receptor for CEA [17, 22]. The PELPK peptide sequence of CEA is important for hnRNP M4 binding [17, 79]. Other proteins that contain the PELPK sequence reacting with hnRNP M4 have not been found.

Kupffer cells can clear circulating CEA in the blood. Notably, liver or lung metastasis from CRC cells begins with the binding with CEA and hnRNP M4-mediated cellular uptake of CEA. Patients who produce PELPK mutant CEA have very high serum CEA levels. In addition, the mutant CEA displays a lower clearance rate from the circulation in experimental animals [79], indicating that PELPK is important in the binding of CEA with hnRNP M4 and cellular uptake in Kupffer cells.

Kupffer cells are activated by the interaction with CEA. The activated cells induce the overexpression of cytokines and change the microenvironment to allow circulating colorectal tumor cells to survive in the liver [80]. Activated Kupffer cells produce a series of cytokines, chemokines, proteins, and metabolites. These include interleukin- (IL-) 1-*α*, IL-1-*β*, IL-6, and IL-10; interferon-*γ* (IFN-*γ*); TGF-*β*; TNF-*α*; platelet-activating factor (PAF); monocyte chemotactic protein-1 (MCP-1); macrophage inflammatory protein (MIP-1); matrix metalloproteinase- (MMP-) 1, MMP-7, and MMP-13; oxygen and nitrogen species including superoxide, hydrogen peroxide, and nitric oxide; and the lipid metabolites prostaglandin D2 and E2 [24, 25, 81, 82]. Interleukins and TNF-*α* are especially important cytokines for Kupffer cell activation. Their production in localized microenvironments within the hepatic sinusoid has various biological effects [83].

Cell adhesion is critical for circulating tumor cells to be arrested and to survive in distant secondary organs. Kupffer cells that are activated upon binding of CEA can produce IL-1-*β* and TNF-*α* which can increase the adhesion of CRC cells to endothelial cells [24, 25, 81–84]. Generation of cytokines from human Kupffer cells results in the overexpression of cell adhesion molecules such as ICAM-1, VCAM-1, and E-selectin in endothelial cells which can be detected in a multicell coculture system incubated with CEA-producing colon cancer cells, Kupffer cells, and endothelial cells [26, 84].

Arrest of circulating tumor cells in the liver can increase the production of nitric oxide (NO) and reactive oxygen species (ROS) to remove tumor cells. NO and ROS have important roles in macrophage-mediated immunity [85]. They affect cancer-related cellular functions like cell survival, intravasation, and angiogenesis. Regulation of the level of NO is a clinically important means to control cancer progression [86]. NO and ROS adversely affect the liver, resulting in immune response-mediated death of CRC cells [87]. CEA-activated Kupffer cells can release the anti-inflammatory cytokine, IL-10, that is important in tumor cell survival due to the inhibition of the upregulation of inducible nitric oxide synthase and the production of NO and ROS [26, 27]. IL-6 secreted by activated Kupffer cells can promote metastasis through hepatocyte growth factor (HGF) [88]. A correlation between the expression level of CEA and IL-6 has been reported in the serum of CRC patients [89].

Liver metastasis in CRC necessitates completion of a series of steps. Circulating CRC cells expressing CEA can block anoikis in the blood. They can encounter Kupffer cells in the liver and change the liver microenvironments to favor establishment of a tumor. How CEA affects the multiple steps of liver metastasis, such as how CEA interaction with Kupffer cells induces signal transduction and activates cells, remains unclear.

### 3.3. Activation of Cell Adhesion-Related Proteins by CEA

Cell-to-cell adhesion is critical for communication with neighboring cells and tissue architectures. CEA that is present as a GPI-linked membrane-anchoring protein functions as a cell-to-cell adhesion molecule connecting epithelial cell membranes and in cell clustering [9]. GPI-linked CEA also affects intercellular adhesion through antiparallel reciprocal self-interaction. CEA functions as a cell adhesion molecule through the CEA-to-CEA homophilic interaction or CEA to CEACAM1 or by CEACAM6 heterophilic interaction. For homophilic and heterophilic interactions, an interaction of the N domain with variable Ig domains and the A3B3 domain of the counterpart CEA is required [90, 91]. This phenomenon is a unique characteristic of CEA [35, 36]. Although the functions of the A3B3 domain are not been fully characterized, 28 asparagine-linked highly glycosylated sites are present in the A3B3 domain [92]. Whether these modifications are important in the antiparallel reciprocal self-interaction is unclear.

Cell surface CEA disrupts tissue architecture and inhibits differentiation and anoikis through the activation with the integrin signal pathway [8]. In the cell membrane, GPI-linked CEA and *α*5*β*1 integrin colocalize. The *α*5*β*1 integrin is the main receptor of ECM. Thus, its colocalization with CEA increases the binding of cells to fibronectin and activates downstream signals through the regulation of PI3K and AKT activity [93]. N domain deletion mutant of CEA is incapable of self-binding or clustering, whereas the N domain deletion mutant CEA can colocalize with the *α*5*β*1 integrin on the cell surface [93]. These results indicate that the N domain of CEA is dispensable for the colocalization of CEA and *α*5*β*1 integrin.

**Figure 2:**
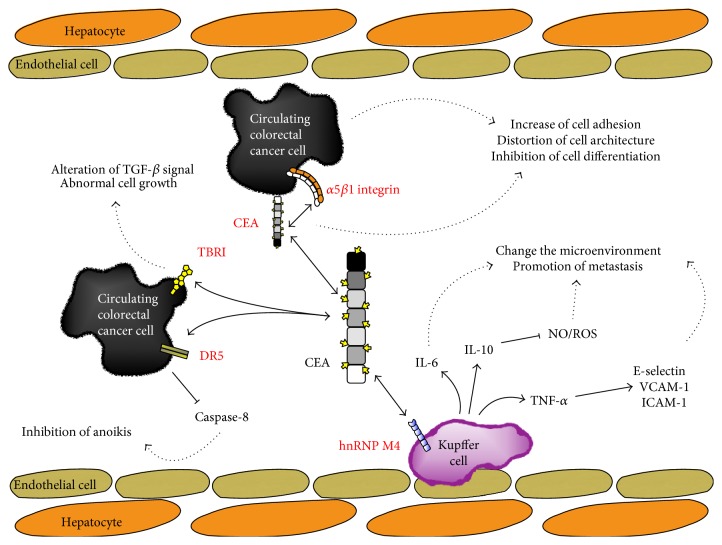
Schematic representation of CEA-affecting biological events. Proteins indicated by the red letters are direct interacting molecules with CEA. Dotted arrows mean consequences of CEA-affecting biological events. In circulating colorectal cancer cells, DR5 (death receptor 5) and TBRI (TGF-*β* type I receptor) interact with CEA. Interaction with DR5 results in the inhibition of caspase-8 activity, inducing anoikis inhibition. Interaction with TBRI makes alteration of TGF-*β* signal pathways. Hence, cancer cells can abnormally overgrow. In Kupffer cells, hnRNP M4 interacts with CEA and secretes a series of cytokines such as IL-6, IL-10, and TNF-*α*. These cytokines can change liver microenvironments to a metastatic friendly environment for efficient metastasis of primary tumor cells. CEA also interacts with CEA antiparallelly and integrin. Interaction of CEA and integrin with CEA increases cell adhesion and distortion of cell architecture while inhibiting cell differentiation.

Glycosylation is one of the most frequent posttranslational modifications of proteins. Glycosylated proteins have critical roles in tumor cells [94]. Glycosyltransferase overexpression is a tumor hallmark and can be used as a tumor marker [95]. Glycosylation-modified CEA is highly expressed in colon cancer tumors compared to that in normal tissues [43]. Tumor-specific glycosylated CEA can interact with dendritic cell-specific intercellular adhesion molecule-3-grabbing nonintegrin (DC-SIGN) [43, 96]. This interaction is mediated through the binding of DC-SIGN with Lewis(x) and Lewis(y), which are present in high levels in CEA of CRC cells [96]. Interaction of tumor CEA and DC-SIGN might suppress tumor-specific immune responses of dendritic cells for tumor progression. CEA-affected biological events in liver metastasis are summarized in Figure 2.

## 4. CEA-Targeted Therapeutic Approaches

Since the discovery that the overexpression of CEA is strongly associated with CRC progression and liver metastasis, targeting of CEA as an anticancer therapeutic approach has been attempted. Diverse tools targeting CEA have been developed and clinically explored; these include vaccines [97–101], dendritic cells [102–104], and antibodies [105–114].

Vaccines have been developed most intensely. Immunization with vaccinia virus expressing recombinant CEA can significantly reduce the growth of tumors transduced with the CEA gene in a syngeneic mouse model [101]. Vaccines have been designed to induce immune responses against tumor-specific antigens or tumor-associated antigens, with the aim of inhibiting the progression of cancers expressing either antigen. Engineered viruses or DNA vectors expressing CEA have been developed as vaccines to induce immune responses against CEA-expressing cancer cells [97–100]. Dendritic cell-based vaccines have also been developed by loading dendritic cells with CEA peptide or mRNA to induce CEA-specific T cell responses [100, 102, 103]. Recombinant virus- or DNA-based and dendritic cell-based vaccines have shown a strong immune response to CEA, resulting in delayed tumor progression and prolonged survival in some cancer patients [104]. However, in that study, vaccination failed to remove tumors in most cases probably due to the inhibitory effect of tumor microenvironment on immune response. Therefore, cotreatment with drugs that can hamper the immunosuppression effects is needed to optimize the effect of cancer vaccines.

CEA-specific antibody-based approaches have also been intensely studied to inhibit cancer progression. Diverse target-specific antibodies have been developed as drugs. Many of them, especially those against cancer and rheumatism, are already commercially available and popular. CEA-specific antibodies can efficiently inhibit cancer progression and metastasis in animal models [105]. Treatment of CEA-specific antibodies alone has shown minimal effects in clinical trials most probably due to their poor tumor penetration and rapid clearance of high-affinity antibodies with free circulating CEA [115, 116]. To enhance the anticancer effects, CEA antibodies have been conjugated with various molecules, such as radioisotopes, immunotoxins, cytokines, and cytotoxic enzymes [117–120]. Recently developed combinatorial regimens utilized CEA and T cell bispecific antibodies or bispecific antibodies combined with antibodies against immune checkpoint molecules including anti-PD-1 or anti-PD-L1 antibodies [109, 110]. The combinatorial approaches showed more effective anticancer effects in clinical study by maximizing the recruitment of T cells and killing of tumors.

Despite the diverse tool-based CEA-targeting anticancer approaches, tumor-targeting and tumor-suppressing activities are still limited. Most effective CEA-targeting tools are based on the induction of an immune response against CEA. In contrast, few drugs directly target CEA have been developed. A new therapeutic approach against liver metastasis was developed through the identification of an antimetastatic, CEA-specific RNA aptamer [70]. An aptamer is a single-stranded DNA or RNA nucleic acids that can bind to specific molecular targets that include proteins, chemicals, ions, and cells. It can be identified by in vitro selection methods termed systematic evolution of ligands by exponential enrichment (SELEX) [121]. Aptamers have therapeutic advantages compared to antibodies, including small size, high affinity and specificity, penetration to tumor tissue, efficient chemical synthesis and conjugation, and low immunogenicity [122]. Aptamers also have advantageous characteristics in cancer therapy and imaging, including rapid tumor uptake, rapid blood clearance, and long-term tumor retention [123]. CEA-specific aptamer can specifically bind to the aforementioned PELPK penta-peptide amino acids, which have important roles in liver metastasis and anoikis resistance. Importantly, an aptamer can efficiently reduce the volume of hepatic metastatic tumor from colon cancer cells in mouse models [70]. Binding of a CEA aptamer with the PELPK penta-peptide sequence can inhibit the binding ability of CEA with hnRNP M4 or DR5, which blocks liver metastasis and bestows anoikis sensitivity to cancer cells. Moreover, aptamer-specific binding with CEA protein and CEA-expressing cell surface can be useful as a cell-targeting and capturing, diagnostic, and molecular imaging tool. Anoikis resistance of circulating cancer cells caused by upregulation of CEA can induce drug resistance [113, 124]. Therefore, CEA-targeting aptamer used alone or in combination with chemotherapeutic drugs, such as mixture of aptamer and drugs or aptamer-drug conjugates, can be a beneficial modality against metastasis.

## 5. Conclusion

Metastasis is a major hurdle to overcome in curing cancers. In contrast with primary cancers, metastasis-mediated spread of secondary cancers is difficult to eradicate. Moreover, they recur easily. A correlation between CEA expression in cancer cells and metastasis has been confirmed. The PELPK region between the N and A1 domains of the hinge region of CEA is important in liver metastasis from CRC. It protects circulating CRC cells from anoikis in blood vessels and orchestrates the functions of Kupffer cells to change the liver microenvironments into a metastatic friendly environment, aiding the survival of cancer cells in the liver.

CEA overexpression is associated with many types of cancers. CRC patients display high expression levels of CEA. CEA is used as a tumor marker after cancer therapy or surgery in cancer patients. Monitoring and measurement of circulating CEA level is useful in patient prognosis and diagnosis. Since CEA is a clinically meaningful target due to its strong correlation with cancer progression, metastasis, and drug resistance, diverse clinical attempts are ongoing to develop various tool-based CEA-targeting anticancer drugs.

Details of how CEA aids liver metastasis from circulating CRC cells remain vague. Nevertheless, accumulating experimental data have indicated the important roles of CEA in metastasis and tumorigenesis. Further understanding of detailed functions, mechanisms, and regulation of CEA are anticipated to lead to the development of more effective modalities against cancer.
